# The antibacterial, phytochemicals and antioxidants evaluation of the root extracts of *Hydnora africana*Thunb. used as antidysenteric in Eastern Cape Province, South Africa

**DOI:** 10.1186/s12906-015-0835-9

**Published:** 2015-09-04

**Authors:** OA Wintola, AJ Afolayan

**Affiliations:** Medicinal Plants and Economic Development Research Centre, Department of Botany, University of Fort Hare, Alice, 5700 South Africa

**Keywords:** *Hydnora africana*, Medicinal plants, Antibacterial, Phytochemical, Antioxidant, South Africa

## Abstract

**Background:**

To determine the anti-dysenteric, phytochemicals and antioxidative properties of the root extracts of *Hydnora africana.* The use of plants for the treatment of dysentery and other diseases in traditional medicine has increased on the basis of these rich traditional medicine systems. Series of pharmacological tests are recommended since the aetiology of many diseases may be due to more than one factor.

**Methods:**

The agar well diffusion method was used to determine the susceptibility of bacterial strains to crude extracts of the plant. The minimum inhibitory concentration was determined by the microdilution test. The presence of phytochemicals and antioxidant was also assessed using standard methods.

**Results:**

The antimicrobial activity of *H. africana* against all the tested organisms demonstrated a mean zone diameter of inhibition ranging from 0 to 25 mm. The MIC of the extracts ranged from 0.071 to 5.0 mg/mL. Antioxidant activity showed lower ferric reducing activities, moderate nitric oxide, moderate DPPH and higher ABTS scavenging activities of the plant. Phytochemical assay revealed the presence and equivalent quantity of alkaloids, tannins, flavonoids, saponins and phenolic acid in the extracts. The water and methanol extracts were also shown as the best solvents of extraction for the phytochemicals.

**Conclusions:**

The methanol and acetone extracts of *H. africana* exhibited a significant antibacterial and antioxidant activities, suggesting the presence of either good bioactive potency or the high concentration of the active principle in the extracts which may serve as a guide for selecting bio- medicinal substances of plant origin in antidysenteric drugs.

## Background

The reliance on natural plants for the treatment of various diseases has increased globally on the basis of their rich traditional medicine systems mostly in the rural area where western doctors are outnumbered by native medicine practitioners [1, 2]. It is believed that about 25 % of most prescribed drugs are obtained from plants. This is evidence in the WHO’s report on the essential usage of plants drugs where 11 % from 252 plant species were alleged as being used as drugs currently [3, 4]. The therapeutic use of herbal remedies has served as important source of new drugs, new drug leads and new chemical entities with about 350,000 plant species identified, most of which are yet to be investigated for their pharmacological and phytochemical potentials [4].

The ever-increasing use of medicinal plants has resulted in the over exploration of these natural resources from plants and has made some South African indigenous plants to be at the risk of becoming extinct before their therapeutic potentials are investigated. The various therapeutic potentials of plants need to be investigated to prevent the erroneous eradication of vital information which could serve as a lead for effective drug development.

As a result, a twofold screening approach of medicinal plants has been recommended for fast discovery of plants' derived bioactive compounds, considering the low and different quantities of chemicals in plants [4, 5]. Series of pharmacological tests are recommended to locate the active compounds as well as to determine the biochemical activities of the plant and the mechanisms of action since the aetiology of many diseases is frequently due to more than one factor [6].

Plant biomolecules have been reported to be alternatives to antibiotics resistance of human pathogens because of their proven effectiveness and availability [7]. Human pathogens are able to cause diseases such as dysentery which has been reported as one of the most serious infectious bacterial diseases, causing a threat to healthcare globally, despite the availability of drugs and care centres. Diarrhea diseases could be caused as a result of oxidative diseases, AIDS, organ transplant, cancer therapy as well as aging which often increased microbial infections [8]. Due to the important role medicinal plants play in the process of drug discovery and development, they are widely recognised as sources of active antimicrobial as well as antioxidant metabolites [9]. These metabolites are important mediators of provocative processes causing chronic diseases such as cancer, inflammation, cardiovascular as well as bacterial and viral diseases [8, 10].

The uses of chemical compounds found in the various plant species have different medicinal effects which have been shown to have scientific basis [4]. The biomolecules (phytochemicals) help the body cell wall and DNA to reduce and neutralize reactive oxygen species (ROS) such as hydroxyl (OH^−^), superoxide (O_2_^−^), nitric oxide (NO), peroxyl (RO_2_^−^), lipid peroxyl (LOO^−^). generated during normal metabolic processes in human body.

*Hydnora africana* Thumb. known also as Jackal food or Jakkalskos, Ubnklunga (X) Umavumbuka (Z) Umafumbuka (X) is one of about ten species in the parasitic flowering plant Hydnoraceae (Piperales). These unusual plants live most or all of their life cycle underground and do not make chlorophyll or have the ability to photosynthesize [11]. The root, fruits, tuber, leaves and fruit pulp (like potato) are used in folk medicine to treat infectious-related diseases such as diarrhoea, dysentery amenorrhoea, poor kidney and bladder conditions. Other conditions include swollen glands and inflamed throat. The plant is also used as a tanning agent for fishing nets. The antimicrobial potentials of this plant for modern medicines have been reported [12–17]. Despite the folklore use of this plant, there is a dearth of scientific information on its phytochemical, antioxidant and antidysenteric activities. Hence, the present study seeks to examine this valuable indigenous medicinal plant based on its local uses as a treatment for dysentery with the hope of determining the phytochemical composition of the plant and to carry out microbiological activity tests so as to see whether the growth of organisms, known to be the causative agents of dysentery, could be inhibited.

## Methods

### Plant material

Fresh mature whole plant of *Hydnora african* was collected in December, 2013 at Ntselamanzi area of the Eastern Cape Province of South Africa. The plant material was authenticated by Prof DS Grierson, a botanist in the University of Fort Hare, Alice South Africa. Specimen sample (Win 2014/1) was prepared and deposited at the Giffen's herbarium.

### Extraction methods

The powdered plant material (200 g) was oven dried to constant weight at 40 °C, milled to a homogeneous powder and extracted separately in distilled water, acetone and methanol on a shaker (Orbital Incubator Shaker, Gallenkamp) at 140 rev/min) for 48 h with. The extract was filtered using a Buchner funnel and Whatman No. 1 filter paper. The filtrate obtained with water was frozen at −40 °C and dried for 48 h using a freeze dryer (Vir Tis benchtop K, Vir Tis Co., Gardiner, NY). The extracts were further concentrated to dryness under reduced pressure at 37 °C using a rotary evaporator (Strike 202 Steroglass, Italy) to remove the solvents. The resulting extracts were reconstituted with their respective solvents to give the desired concentrations used in the study.

### Antibacterial assay

#### Test organisms

Reference bacterial strains were obtained from the Department of Biochemistry and Microbiology, University of Fort Hare, Alice, South Africa, which included Salmonella typhimurium (ATCC 13311), Enterococcus faecalis (ATCC 29212), Escherichia coli (ATCC 25922); Pseudomonas aeruginosa ATCC 19582, Bacillus cereus (ATCC10702), Shigella sonnei ATCC 29930, Streptococcus pyogens, Bacillus subtilis KZN, Shigella flexneri KZN, Vibrio cholerae (Laboratory isolate), Klebsiella pneumoniae ATCC 4352, Staphylococcus aureus (ATCC 6538). The strains were kept at 4 °C on agar slant and sub cultured at 37 °C for 24 h on nutrient agar before any susceptibility test. The antibacterial assays were carried out using Nutrient Agar (Biolab) and broth.

#### Antibacterial susceptibility test

The agar well diffusion technique was employed as previously described by Otang et al. [18] with some modifications. Using the micropipette, 100 μl of 0.5 McFarland solutions of bacterial strain cultures in 0.9 % sterile distilled water (SDW) was dispensed over the surface of an agar plate and spread using a sterile inoculation loop. Four wells were cut in each agar plate with a heat sterilised cork borer of 6 mm diameter, and the agar plugs removed with a sterile needle. In the first hole, 50 μl of a positive control drug was added (Ciprofloxacin 0.0125 mg/ml); 50 μl of the acetone, methanol and aqueous solvent was added as a negative control in the second hole; and 50 μl of the plant extract was added in the third and last holes at concentrations of 25 and 50 mg/ml, respectively. Each test was duplicated. The culture plates were then incubated at 37 °C, and the results were observed after 24 h. The clear zone around each well indicating the activity of the plant extract against the bacterial organisms was measured in mm.

#### Micro dilution assay

The micro dilution method was employed to determine the minimum inhibitory concentration (MIC) of the plant extracts using 96 well microtitre plates [19]. Initially, 120 μl of SDW was added into each well of the first (A) and last (H) rows and also into all the wells of the last column [20]. Then, 120 μl of Nutrient broth (NB) was added into each well of the second row (B) and 150 μl of NB was added into the remaining wells of the first column and 100 μl into the rest of the wells from the second column rightward. Fifty microlitres of the plant extract were then added into the third well of the first column while 50 μl of the positive and negative control were separately added into the remaining wells of the first column.

A two-fold serial dilution was done by mixing the contents in each well of the first column (starting from the third row) and transferring 100 μl into the second well of the same row and the procedure was repeated up to the 11^th^ well of the same row and the last 100 μl from the 11th well was discarded. Hence various concentrations of the plant extracts ranging from 5 mg/ml to 0.005 mg/ml were prepared in the wells, following the two-fold dilution method. Thereafter, 20 μl of 0.5 McFarland bacteria suspensions was inoculated into the wells except those which contained SDW. The growth of the bacteria was measured by determining the absorbance at 620 nm with a microtitre plate reader before and after incubation. The plates were incubated at 37 °C for 24 h. The lowest concentration of the test extract resulting in inhibition of 50 % of bacterial growth was recorded as the MIC.

### Phytochemical

#### Determination of total phenols

The Folin - Ciocalteu assay described by Wintola and Afolayan [21] was used for the quantification of total phenolic content. Absorbance at 765 nm was read using an AJI-C03 UV–VIS spectrophotometer after incubating the reaction mixtures at room temperature for 30 min. The assay was done in triplicate. Results were expressed as mg/g of tannic acid equivalent using the calibration curve: Y = 0.1216x, R^2^ = 0.936512, where Y was the absorbance and x was the tannic acid equivalent.

#### Estimation of flavonoids

Flavonoid content was measured using the aluminum chloride colorimetric method as described by Oyedemi et al. [22]. Absorbance at 420 nm was read using an AJI-C03 UV–VIS spectrophotometer. The samples were analyzed in triplicate, total flavonoids content was calculated as mg/g of quercetin using the following equation based on the calibration curve: Y = 0.0255x, R^2^ = 0.9812, where Y was the absorbance and x quercetin equivalent.

#### Determination of total proanthocyanidin

Proanthocyanidin content was measured using the vanillin-methanol procedure described by Oyedemi et al. [22]. Absorbance at 500 nm was read using an AJI-C03 UV–VIS spectrophotometer against a blank that contained 50 % aqueous methanol. Total proanthocyanidin content was evaluated at a concentration of 0.1 mg/ml and expressed as catechin equivalent (mg/g) using the calibration curve equation: Y = 0.5825x, R^2^ = 0.9277, where Y was the absorbance and x is the Catechins equivalent.

#### Tannin determination

Folin-Denis reagent tannin determination was done using the methods described by Mbaebie et al. [23]. The absorbance of the tannic acid standard solutions as well as sample was measured after color development at 760 nm using the AJI-C03 UV–VIS spectrophotometer. Results were expressed as mg/g of tannic acid equivalent using the calibration curve: Y = 0.0593x – 0.0485, R^2^ = 0.9826, where Y was the absorbance and x was tannic acid equivalent.

#### Determination of saponins

Quantitative determination of saponins was done using the method of Okwu and Josiah, [24].

Five grams of plant sample was dispersed in 50 ml of 20 % v/v aqueous ethanol. The suspension was heated over hot water bath for 4 h with continuous stirring at 55 °C. The mixture was filtered and the residue re-extracted with another 50 ml of 20 % aqueous ethanol. The combined extracts were reduced to 20 ml over hot water bath at about 90 °C. The concentrated solution obtained was shaken vigorously with 10 ml of diethyl ether in a 250 ml separating funnel. The aqueous layer was collected while the ether layer was discarded. The purification process was repeated. Twenty millilitre of butanol was added to the filtrate and then washed twice with 10 ml of 5 % w/v aqueous sodium chloride. The whole mixture was heated to evaporation on hot water bath and later oven dried at 40 °C to a constant weight. The saponins content was calculated using the formula: % saponins = final weight of sample/initial weight of extracts × 100.

#### Determination of alkaloids

Alkaloids were quantitatively determined according to the method of Wintola and Afolayan, [21]. Five (5 g) of plant sample was extracted in 200 ml of 10 % acetic acid in ethanol. This was allowed to stand for 4 h at room temperature and filtered, the filtrate was then concentrated on a water bath to ^1^/_4_th of its original volume. Concentrated ammonium solution was added drop wise to basify the extract until the precipitation was completed and the whole solution was allowed to settle. The collected precipitates were washed with diluted ammonium solution and then filtered. The residue was dried and weighed. The alkaloid content was determined using this formula: % Alkaloid = final weight of sample/initial weight of extract × 100.

### Antioxidant assay

The antioxidant activities of the whole plant extracts of *H. africana* were determined using DPPH, ABTS, reducing power and nitric oxide.

#### Determination of ferric reducing power of the extracts

The reducing power of the whole plant extract of *H. africana* was evaluated according to the method described by Aiyegoro and Okoh [25]. The mixture containing 2.5 ml of 0.2 M phosphate buffer (pH 6.6) and 2.5 ml of K_3_Fe (CN)_6_ (1%w/v) was added to 1.0 ml of the extracts and standards (0.025-0.5 mg/ml) prepared in distilled water. The resulting mixture was incubated for 20 min at 50 °C, followed by the addition of 2.5 ml of trichloroacetic acid (10 % w/v), which was then centrifuged at 3000 rpm for 10 min. 2.5 ml of the supernatant was mixed with 2.5 ml of distilled water and 0.5 ml of FeCl_3_ (0.1 %, w/v). The absorbance was then measured at 700 nm against blank sample. Increased absorbance of the reaction mixture indicated higher reducing power of the plant extract.

#### DPPH radical scavenging assay

The reducing power of the whole plant extract of *H. africana* was evaluated according to the method described by Aiyegoro and Okoh [25] was used for the determination of scavenging activity of DPPH free radical. DPPH (1 ml, 0.135 mM) prepared in methanol was mixed with 1.0 ml of extracts ranging from 0.025-0.5 mg/ml. The reaction mixture was vortexed thoroughly and left in the dark at room temperature for 30 min. The absorbance was measured spectrophotometrically at 517 nm. The scavenging ability of the plant extract was calculated using this equation: DPPH Scavenging activity(%) = [(Abs control – Abs sample)]/(Abs control)] × 100, where Abs control is the absorbance of DPPH + methanol; Abs sample is the absorbance of DPPH radical + sample (sample or standard).

#### ABTS radical scavenging activity

The method described by Adedapo et al. [26] was adopted for the determination of ABTS activity of the plant extract. The working solution was prepared by mixing two stock solutions of 7 mM ABTS and 2.4 mM potassium persulphate in equal amounts and allowed to react for 12 h at room temperature in the dark. The resulting solution was further diluted by mixing 1 ml ABTS. + solution with 60 ml methanol to obtain an absorbance of 0.706 ± 0.001 units at 734 nm after 7 min using spectrophotometer. The percentage inhibition of ABTS. + by the extract was calculated from the following equation: % inhibition = [(Abs control – Abs sample)]/(Abs control)] × 100.

#### Nitric oxide scavenging activity

The modified method described by Oyedemi et al.[22] was used to determine the nitric oxide radical scavenging activity of aqueous and other solvent extracts of *H. africana*. A volume of 2 ml of 10 mM of sodium nitroprusside (Na_2_Fe(CN)2NO.2H_2_0) prepared in 0.5 mM phosphate buffer saline (pH 7.4) was mixed with 0.5 ml of plant extracts, gallic acid and BHT individually at 0.025-0.5 mg/ml. The mixture was incubated at 25 °C for 150 min. 0.5 ml of incubated solution was mixed with 0.5 ml of Griess reagent [1.0 ml sulfanilic acid reagent (0.33 % prepared in 20 % glacial acetic) acid at room temperature for 5 min with 1 ml of naphthylenediamine dichloride (0.1 % w/v)]. The mixture was incubated at room temperature for 30 min, followed by the measurement of the absorbance at 540 nm. The amount of nitric oxide radicals inhibited by the extract was calculated using the following equation; NO radical scavenging activity (%) = [(Abs control – Abs sample)]/(Abs control)] × 100, where; Abs control is the absorbance of NO radicals + methanol; Abs sample is the absorbance of NO radical + extract or standard.

## Results

### Antibacterial

The antibacterial activities of plant extracts have been linked to the presence of some bioactive compounds or secondary metabolites. These secondary metabolites provide protection to the plants themselves against bacterial, fungal and viral infections. Among the three extracts that were tested against the 12 pathogenic bacteria, methanol and acetone extracts were active against at least one of the bacteria with zones of inhibition varying from 12 to 20 mm (Table 1). Aqueous extract was not active against any of the twelve bacteria tested. The acetone extract was active against all the bacteria the tested bacteria, while the methanol extract was active against all bacterial except *Salmonella typhimurium.* The inhibitory activity of the extracts based on the overall mean inhibition diameters in the order: acetone > methanol > aqueous.

**Table 1:** Average zone of inhibition (±1.00 mm) produced by different *H. africana* extracts used for the management of dysentery at different concentrations and ciprofloxacin against the selected bacterial strains

Bacteria	Zones of inhibition			
	Aqueous	Ethanol	Acetone	Positive control (Ciprofloxacin)
*Salmonella typhimurium*	NA	NA	10 ± 1.1	25 ± 1.1
*Enterococcus faecalis*	NA	15 ± 1.0	16 ± 1.2	24 ± 1.2
*Escherichia coli*	NA	20 ± 0.1^a^	15 ± 2.1	25 ± 0.2
*Pseudomonas aeruginosa*	NA	20 ± 1.1^a^	15 ± 1.3	24 ± 1.1
*Bacillus cereus*	NA	15 ± 1.2	15 ± 1.2	24 ± 1.2
*Shigella sonnei*	NA	15 ± 0.2	20 ± 2.2^a^	24 ± 2.2
*Streptococcus pyogens*	NA	15 ± 0.2	13 ± 1.2	23 ± 2.1
*Bacillus subtilis*	NA	15 ± 1.1	15 ± 0.5	22 ± 0.2
*Shigella flexneri*	NA	15 ± 0.3	16 ± 0.5	24 ± 1.1
*Vibrio cholerae*	NA	16 ± 2.0	16 ± 0.6	20 ± 1.3
*Kleb pneumoniae*	NA	15 ± 0.6	14 ± 1.3	24 ± 2.2
*Staphylococcus aureus*	NA	12 ± 0.8	15 ± 0.4	24 ± 0.2

^a^Not statistically different from the positive control; NA, not active

The highest activity against the tested bacteria was obtained with the methanol extract with inhibition zones diameters of 20 mm against *Escherichia coli* and *Pseudomonas aeruginosa* similarly the acetone extract produced a highest activity with inhibition zones diameters of 20 mm against *Shigella sonnei* (Table 1). The lowest activity was obtained with the acetone extract having inhibition zones of 10 mm in diameter against *Salmonella* typhimurium, while *Salmonella* typhimurium was not susceptible to the methanol extract. Ciprofloxacin (0.0125 mg/ml), produced a higher inhibition zone of 25 mm against *Salmonella typhimurium* and *Escherichia coli* and lowest of 20 mm against *Vibro cholerae*.

The varying concentrations between 5 and 0.005 mg/ml of the plant extracts were tested in order to determine their MICs. The MICs of the plant extracts against the 12 tested bacterial is presented in Table 2. The lowest MICs were obtained in the methanol extract having 0.01 mg/ml against *E. coli*, 0.02 mg/ml against *P. aeruginosa* and 0.08 mg/ml against *S. pyogens*. And the acetone extract had 0.08 mg/ml against *S. typhimurium*, 0.16 mg/ml against *B. cereus*, and 0.02 mg/ml against *Shigella sonnei*. The aqueous extract was inactive against all the bacterial isolates at the concentration tested (Table 2). The MIC ranged from 0.01 to 5 mg/mL for all studied microorganisms while for ciprofloxacin it ranged from 0.01 to < 0.10. These extracts containing various phytochemical compounds are therefore known to be biologically active as well as showing antimicrobial activities. In this study, the Gram negative bacterial are more susceptible than the Gram positive ones.

**Table 2:** Minimum inhibitory concentration (mg/ml) of different *H. african* extracts used for the management of dysentery against the selected bacterial strains

Extract	Bacteria											
	*Salmonella typhimurium*	*Enterococcus faecalis*	*Escherichia coli*	*Pseudomonas aeruginosa*	*Bacillus cereus*	*Shigella sonnei*	*Streptococcus pyogens*	*Bacillus subtilis*	*Shigella flexneri*	*Vibrio cholerae*	*Klebsiella pneumonia*	*Staphylococcus aureus*
Aqueous	Na	Na	Na	Na	Na	Na	Na	Na	Na	Na	Na	Na
Methanol	Na	2.5	0.01^a^	0.02^a^	0.16	2.5	0.08^a^	2.5	2.5	2.5	2.5	5
Acetone	0.08^a^	2.5	2.5	2.5	0.16	0.02^a^	0.625	2.5	2.5	2.5	2.5	2.5
Positive control	0.01	<0.1	<0.01	0.01	<0.01	<0.01	<0.01	0.02	<0.02	<0.01	<0.01	<0.1

^a^Not statistically different from the positive control; NA, not active

#### Phytochemicals

**Fig. 1:**
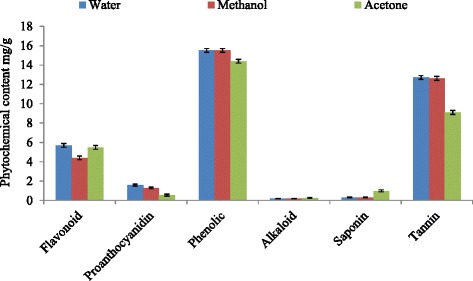
Phytochemicals of the various solvent extracts of H. africana root and the standards

Phytochemical compounds are known to be biologically active and thus aid antimicrobial activities of plants. The phytochemical activities of *H. africana* extracts reveal the presence of alkaloids, saponins, tannins, anthocyanins, flavonoids and phenolic acid. The solubility of these phytochemicals in different solvents was evident in the tannin concentration which ranges from 12.71 to 9.1 mg/g. The concentration of the phenol and tannin was higher than those of other phytochemicals as shown in Fig. 1.

The ranges of the solubility of the plant in different solvents was not statistically different in total flavonoids (5.70 to 4.40 mg/g), total proanthocyanidin (0.05 to 1.60 mg/g), total phenol (14.40 to15.50 mg/g), alkaloids (0.19 to 0.26 mg/g), saponins (0.31 to 1.00 mg/g) and tannins (9.10 to 12.71 mg/g) at 95 % confidence interval. The solvent capacity followed the decreasing order of water > methanol > acetone. Water extracted more of the phytochemicals indicating differences in the extracting capacity of the solvents. This showed that water extracted more of the phytochemicals than the organic solvents.

The quantity of phytochemicals extracted from the plant sample revealed the vital role played by the solvent of extraction in the yield. The best solvent of extractions of *H. africana* phytochemicals, therefore, is aqueous followed by methanol and the least is acetone. The concentrations of proanthocyanidin observed in the different solvent extracts of *H. africana* are low (ranging from 0.05 to 1.60 mg/g).

### Antioxidant

**Fig. 2:**
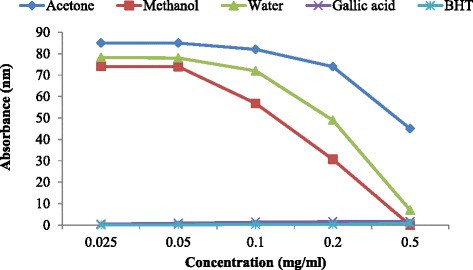
Reducing Power of the various solvent extracts of H. africana and the standards

The reducing power from the different solvent extracts of *H. africana* was evaluated in Fig. 2. All the extracts showed that the ferric reducing activities of the extracts were significantly lower than those of the standard drugs used in this order: gallic acid > BHT > methanol > aqueous > acetone extracts, though the reducing activities of the extracts were significantly lower (P < 0.05) than those of the standard controls. The dose dependent reducing potentials of the extracts suggested that there is the presence of antioxidant compounds with electron-donating ability. The values of the aqueous and methanol extracts were comparable with gallic acid and BHT at 0.5 mg/ml.

**Fig. 3:**
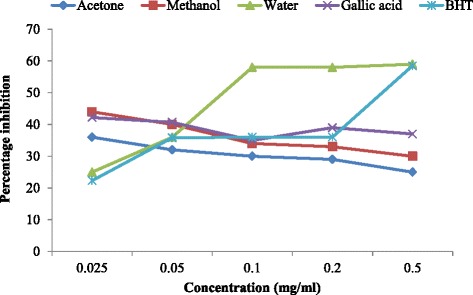
Nitric Oxide of the various solvent extracts of H. africana and the standards

The nitric oxide scavenging activity of the different solvent extract of *H. africana* was comparable with those of the standard controls at all the concentration tested (Fig. 3). Although, the percentage nitric oxide inhibition of the water extract increased rapidly from 20 % to 60 % at the concentration of 0.025 to 0.05 mg/ml, this became constant as concentration increases to 0.1 mg/ml whereas the percentage inhibition for both methanol and acetone extract decreases as the concentration increases. The water extract increased more than the standards, hence water extract scavenge more of the radicals than gallic acid.

**Fig. 4:**
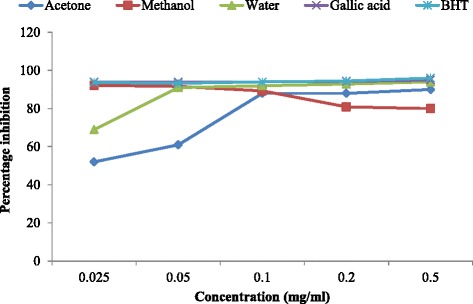
DPPH of the various solvent extracts of H. africana and the standards

The DPPH scavenging ability of the extract of *H. africana* is in the following order methanol > water > acetone. The observed concentration-dependent increase in the absorbance of reaction mixture for all the solvent extracts and the standard drugs (BHT and gallic acid) was illustrated in Fig. 4. At the lowest concentration of 0.025 mg/ml methanol extract was comparable with the standards drugs. At 0.05 mg/ml, the methanol and water extracts were both comparable with the standard while at 0.1- 0.5 mg/ml, all the extracts were comparable with the standard drugs.

**Fig. 5:**
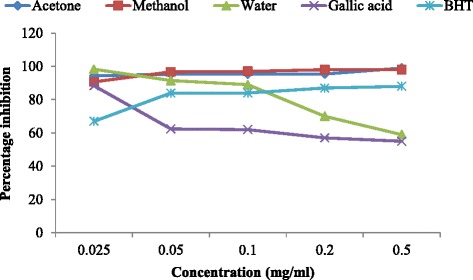
ABTS of the various solvent extracts of *H. africana* and the standards

The ABTS radical scavenging activity of *H. africana* was illustrated in Fig. 5. There was an increase in the radical inhibition of the acetone, methanol and water extracts when compared with the standards at the concentration of 0.025 mg/ml. However, there was no significant difference between the extracts and gallic acid at 0.05 mg/ml. The water extract decreased activity dependently from 0.1-0.5 mg/ml and was not significantly different from gallic acid.

## Discussions

The bacterial isolates used in this study were chosen because they are associated with gastrointestinal infections, while solvent was made based on the dependency of biologically active compound on the solvent used in the extraction procedure [27]. Although native medical practitioners primarily use water, plant extracts from organic solvents have been found to give more consistent antimicrobial activity compared to water extract [28].

The highest sensitivity shown by the tested bacteria against the acetone and methanol extracts of *H. africana* could suggest the presence of antibacterial activity in the plant. Several studies have also been reported by other researchers for the antibacterial activities of *H. africana and H. abyssinica*. Natatiele and Ndip [17], reported that *H. africana* showed antibacterial activity against *Aeromonas hydrophila ATCC 35654*, *Staphylococcus aureus NCT 6571*, *Helicobacter pylori (43526 and PE 252C*). Their findings also showed that all the extracts, except water showed strong activity (13 to 22 mm) against *S aureus and A. hydrophila,* while the methanol and ethyl acetate extracts showed activity against *H. pylori (43526 and PE 252C*). In a similar study, Saadabi and Ayoub [15] demonstrated a very potent antibacterial activity of *H. abyssinica* screened against common pathogenic bacteria including *Escherichia coli, Pseudomonas aeruginosa, Bacillus subtilis and Staphylococcus aureus.* The above findings suggest the presence of antibacterial compounds *in H. africana.* Phytochemical compounds such as alkaloids, saponins, tannins, flavonoids and steroids have been known to be biologically active and thus partially responsible for the antimicrobial activities of plants, hence their use in traditional medicine [17]. These compounds have been identified in the genus *Hydnora* [17, 29, 30].

Crude extracts are considered active, if the MIC value of the extract is 0.1 mg/ml or less. However, in this study, only the methanol extract with MIC of 0.01 mg/ml against *E. coli* exhibits this criteria out of the 12 bacterial strains used. This may indicate that the extract does not have high activities in inhibiting the growth of these organisms and it calls for further investigation on the mode of activity of this plant as it is mostly used traditionally [17]. Although the MIC values of the extracts against bacteria were considerably high (0.01-5.00 mg/mL) when compared with Ciprofloxacin (0.01-0.1 mg/ml). The MICs for the methanol and acetone extract were lower than those obtained for the water extract, indicating that the bacteria were more susceptible to the methanol and acetone extracts than the water extract. The results of the present study support the folkloric usage of the plant and suggests that the plant extracts possess certain constituents with antibacterial properties that maybe used as antibacterial agents in new drugs for the therapy of diarrheal infectious diseases caused by common bacterial pathogens.

Most plant extracts are believed to be more active against Gram positive bacteria because the cell wall is easier to penetrate than Gram negative ones, which contain outer membrane with a lipopolysacharide layer that is impermeable to certain antibiotics and antibacterial compounds [31, 32]. These results provide evidence that some medicinal plants might be potential sources of new antibacterial agents even against some resistant strains of micro-organisms. The reason could be that the extracts were able to penetrate the lipopolysacharide layer more readily thereby making them susceptible.

The presence of flavonoids in crude extract of *H. africana* is important since they have been reported to exhibit antimicrobial, anti-inflammatory, analgesic, anti-allergic, antioxidant, antitrypanosomal and antileishmanial properties [33]. The moderate flavonoids content of this plant account for the wide range of biological activities reported about it which is evident in the antimicrobial activity of the plant. Numerous studies have also been carried out on the antioxidant activities of flavonoids and how they can contribute to the treatment of several diseases like inflammatory, oxidative stress, liver dysfunction, microbial infections, cancer and cardiovascular diseases [34]. This indicated that biological and pharmacological effects of flavonoids may depend upon their behaviour as either antioxidants or as prooxidants [35]. The presence of flavonoids in the plants may have exhibited direct antibacterial activity and suppression of bacterial virulence resulting in the antimicrobial activity seen in this study.

The phenolics were the highest phytochemical in this study and have been reported to have antimicrobial, anti-inflammatory and antioxidant activities too [4, 36]. The redox properties of phenolic compounds have been reported to have an underlying effects in antioxidant activity which makes then donate hydrogen ion to reactive oxygen species (ROS). The presence of a moderate content of flavonoids, tannins and phenol in this study may have contributed to the observed pharmacological activity of this plant in their therapeutic potential.

Proanthocyanidin are believed to occur from macromolecular complex (oligomeric) and polymeric flavan-3-ols and has been reported to demonstrate strong antioxidant capacity [4, 37]. The low value of proanthocyanidin content in all the different solvent extracts of *H. africana* findings suggest that the pharmacological activities of *H. africana* may not be due to proanthocyanidin. The phytoconstituents of various plants have long been known and their antimicrobial properties widely reported [38, 39].

Alkaloid is one of the phytochemical compounds identified in this study. It has been allied with medicinal uses for centuries. Most common biological properties of alkaloids are their toxicity against cells of foreign organisms, anti-inflammatory, anti-asthmatic, and anti-anaphylactic properties [40–42]. The low value of alkaloids content in all the different solvent extracts of *H. africana* suggests that the pharmacological activities of *H. africana* may not be due to the alkaloids.

Saponins are responsible for numerous pharmacological properties and are known to produce inhibitory effects on inflammation [43, 44]. The low value of saponins in all the different solvent extracts of *H. africana* implies that the pharmacological activities of *H. africana* may not be due to saponins.

Tannins exert antimicrobial activities by iron deprivation, hydrogen bonding or specific interactions with vital proteins such as enzymes in microbial cells [45]. Herbs that have tannins are astringent in nature and are used for treating intestinal disorders such as diarrhoea and dysentery [46]. Motar et al. [47] revealed the importance of tannins for the treatment of inflamed or ulcerated tissues. Tannins were observed to have remarkable activity in cancer prevention This is worth noting that *H. africana* could have potentials as a source of important bioactive molecules for the treatment of cancer [48].

The importance of the antioxidant constituents from plant materials is raising interest among scientists, food manufacturers and consumers in the maintenance of health [49, 50]. Antioxidant activities from plants have been attributed to the polyphenolic in plant materials. These antioxidants protect the body against reactive oxygen species (ROS) [51]. The phytochemicals are known to be major plant compounds that have been reported to have multiple biological effects in neutralizing free radicals by donating hydrogen and quenching singlet oxygen [52].

Reducing power is an ionic electron transfer assay characterised by the formation of Perl's Prussian blue coloration after ionic reduction, to produce a reduction in the ferric ion/ ferricyanide complex to ferrous form. The degree of colour change is directly proportional to the antioxidant concentrations in the extracts measured spectrophotometrically [53]. Even though the reducing activities of the extracts were significantly lower than the standard controls (gallic acid and BHT), the dose dependent reducing potentials of these extracts suggest the presence of antioxidant compounds with electron-donating ability. The presence of these compounds in the extracts in an impure form or small amounts may be responsible for the low activity demonstrated by the extracts at 0.025-0.2 mg/ml [4].

### Nitric oxide

The water extract works best at the lowest concentration upward in scavenging the NO free radicals with the highest percentage inhibition of 60 % while the standard gallic acid is very effective at a concentration of 0.2 mg/ml, the methanol and acetone extracts worked best at the lowest concentration (0.025 mg/ml). Hence water extract scavenge more of the radicals than gallic acid. This result conforms with previous studies which reported that aqueous extract showed the highest percentage nitric oxide inhibition at the lowest concentration [49].

The increase in the inhibition of the free radicals indicate that the extracts posses high DPPH scavenging activity even at the lowest concentration. This shows that *H. africana* can serve as a potential antioxidant of natural origin with its effect better than synthetic antioxidants. The reason for the high scavenging activities of the methanol and water extract being more active more than the acetone extract could be as a result of the solvent polarity as reported in our previous work [21].

The percentage inhibition of ABTS radical scavenging of *H. africana* was illustrated in Fig. 5. There was an increase in the radical inhibition of the acetone, methanol and water when compared with the standards at the concentration of 0.025 mg/ml. However, there was a non significant difference between the extracts and gallic acid at 0.05 mg/ml. The water extract decreased from 0.01-0.5 mg/ml and was not significantly different from gallic acid. The increase in the inhibition of the free radicals by the methanol and acetone extracts indicated that the extracts possess high ABTS scavenging activity even at the lowest concentration. This shows that *H. africana* can serve as a potential antioxidant. The reason for the high scavenging activities of the methanol and water extract, being more active than the acetone extract could be as a result of the solvent polarity as reported in our previous work [21].

## Conclusion

In conclusion, the results indicate that the methanol and acetone extracts of *H. africana* exhibited a significant antibacterial activity, suggesting the presence of either good antibacterial potency or the high concentration of the active principle in the extracts. This simply means the plant exhibit moderate antimicrobial activity. The observed tendency of the extract to inhibit Gram-negative bacteria more than the Gram-positive at low concentrations showed that *H. africana* contain interesting bio-medicinal substances capable of attracting significant scientific attention. This study also showed that *H. africana* exhibited antibacterial and antioxidant activities of natural origin which may offer promising antioxidant and antibacterial agents. Investigation will be carried on the *in vivo* antidysenteric activity of the plant and compounds responsible for these activities to be elucidated.
